# Geological CO_2_ quantified by high-temporal resolution stable isotope monitoring in a salt mine

**DOI:** 10.1038/s41598-020-77635-5

**Published:** 2020-11-26

**Authors:** Alexander H. Frank, Robert van Geldern, Anssi Myrttinen, Martin Zimmer, Johannes A. C. Barth, Bettina Strauch

**Affiliations:** 1 Department of Geography and Geosciences, GeoZentrum Nordbayern, Friedrich-Alexander-Universität Erlangen-Nürnberg (FAU), Erlangen, Germany; 2 K+S Aktiengesellschaft, Kassel, Germany; 3 Inorganic and Isotope Geochemistry, Helmholtz Centre Potsdam, GFZ German Research Centre for Geosciences, Potsdam, Germany

**Keywords:** Environmental sciences, Solid Earth sciences

## Abstract

The relevance of CO_2_ emissions from geological sources to the atmospheric carbon budget is becoming increasingly recognized. Although geogenic gas migration along faults and in volcanic zones is generally well studied, short-term dynamics of diffusive geogenic CO_2_ emissions are mostly unknown. While geogenic CO_2_ is considered a challenging threat for underground mining operations, mines provide an extraordinary opportunity to observe geogenic degassing and dynamics close to its source. Stable carbon isotope monitoring of CO_2_ allows partitioning geogenic from anthropogenic contributions. High temporal-resolution enables the recognition of temporal and interdependent dynamics, easily missed by discrete sampling. Here, data is presented from an active underground salt mine in central Germany, collected on-site utilizing a field-deployed laser isotope spectrometer. Throughout the 34-day measurement period, total CO_2_ concentrations varied between 805 ppmV (5th percentile) and 1370 ppmV (95th percentile). With a 400-ppm atmospheric background concentration, an isotope mixing model allows the separation of geogenic (16–27%) from highly dynamic anthropogenic combustion-related contributions (21–54%). The geogenic fraction is inversely correlated to established CO_2_ concentrations that were driven by anthropogenic CO_2_ emissions within the mine. The described approach is applicable to other environments, including different types of underground mines, natural caves, and soils.

## Introduction

Knowledge of geologically derived CO_2_-fluxes in tectonically active and seismic areas, as well as in areas of geothermal heat flow, is well established^1–10^. In the last decade, it has been increasingly acknowledged that the Earth’s mantle and crust CO_2_ emissions may account for a substantial quantity of the global atmospheric carbon cycle^11,12^. This belated recognition might have been rooted in the challenging apportionment of such geogenic CO_2_ contributions. While its analysis is already demanding at active volcanoes, diffusive degassing through the pedosphere involves additional analytical challenges because differentiation from respiration is not easily accomplished by concentration data alone^11,13^.

Nonetheless, diffusive degassing may be the quantitatively predominant process of geogenic CO_2_ losses to the atmosphere^2^. While contributions and their dynamics have been extensively studied near volcanos and in geothermal regions^8,14–18^, short-term temporal and spatial dynamics of geogenic CO_2_-releases to the atmosphere have likely been underestimated^12^, especially from regions without geothermal heat flow. Similar uncertainties exist for natural geogenic CO_2_ emissions from mining environments. Nonetheless, improved inventories of those contributions at locations where biotic components are absent or negligible may help to constrain local geogenic CO_2_ contributions to the aboveground carbon cycle. For this purpose, complex statistical approaches have been applied to distinguish geogenic from biogenic sources^19–21^. Here, we exploited the rare opportunity to access a mine, where geogenic CO_2_ release can be observed directly in several hundred meters depths.

In some specific salt mines, magmatic activity and related CO_2_ may be trapped in salt deposits, from where it can be remobilized by the intrusion of meteoric water along geological faults^22^ or during mining work. Thus, CO_2_ efflux from geological sources is considered a difficult-to-predict threat to mining operations^23^. Such abrupt releases of CO_2_ can easily be attributed to geological sources. However, fluctuations of diffusive CO_2_ influx into mines are subtler. Therefore, they often remain indistinguishable from daily operational fluctuations (caused by machinery and mine venting) when only considering concentrations and discrete samples. Due to significantly different proportions of the two stable isotopes ^13^C and ^12^C in CO_2_ from geogenic and combustion-related sources, this ambiguity can be overcome by stable isotope measurements and the application of isotope mixing models^17,24^.

Here we present a study of underground CO_2_ emissions of a salt mine situated in the Werra-Fulda-mining district (Germany). The potash-bearing salt-deposits of this area are of Upper Permian (Zechstein) origin. During the Oligocene and Miocene, they were influenced by basic magmatism in the Rhön and Vogelsberg^25,26^. Through cooperation with a local mining company, we obtained access to an active mine, where we aimed:

1. To estimate relative flux contributions to the overall CO_2_ present in the mine, and
2. To identify if diffusive geogenic CO_2_ efflux is continuous or shows considerable temporal variabilities.

This was achieved by running a mid-infrared laser isotope spectrometer for several weeks inside the mine at a site where geogenic CO_2_ emissions were suspected due to historical incidents of outgassing, but exact contributions were unknown. This offered an ideal background for mass balance calculations to determine relative proportions of these inputs.

## Results

### Temporal fluctuations

**Figure 1:**
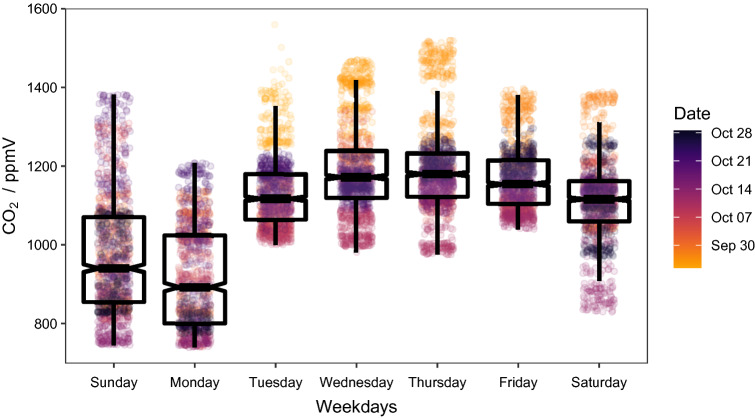
Box and whisker plot of CO_2_-concentrations. Individual measurements (8283) are plotted to show variability. The color scheme corresponds to the date of the measurement, with darker colors showing later sampling.

Total CO_2_ concentrations (C_T_) ranged between 739 and 1560 parts per million with respect to volume fraction (ppmV), with an average of 1102 ppmV, and exhibited strong weekly dynamics (Fig. 1). Days with the highest mean concentrations were Thursdays and Wednesdays. In contrast, Fridays, Tuesdays, Saturdays, Sundays, and Mondays were significantly different from each other and decreased in the above order as indicated by a highly significant ANOVA (F(6, 8275) = 1002, p < 0.001) using Tukey’s HSD (p < 0.001) on heteroskedastic and normal distributed residuals.

These weekly patterns were also confirmed by a highly significant autocorrelation with a 7-day lag (ac = 0.366). A classical additive decomposition approach^27^, applying a symmetrical moving average, was used to differentiate regular weekly, overall trend, and random effects. The regular weekly pattern accounted for 2/3 (67%) of the total observed variation according to a partial linear regression model. Daily variations of the total observed concentration averaged at 223 ± 107 ppmV and ranged between 67 and 591 ppmV.

**Figure 2:**
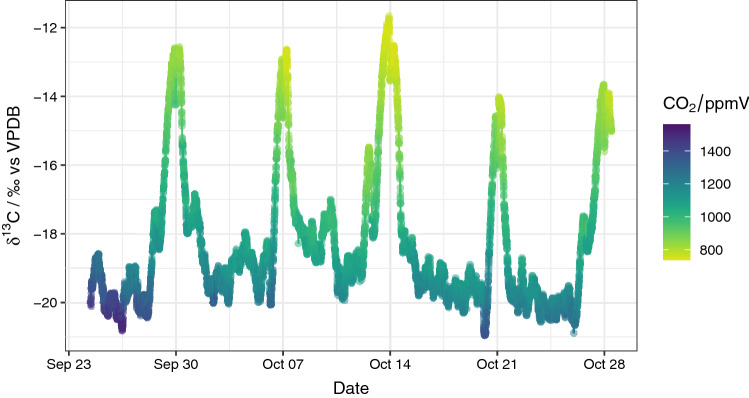
The δ^13^C-CO_2_ isotope composition and concentration over the measurement period. Strong regular temporal variations linked to a 7-day time lag are evident.

Throughout the measurement period, the *δ*^13^C-CO_2_ values fluctuated between − 21.0 and − 11.7‰, with an overall median of − 18.9‰. The *δ*^13^C-CO_2_ is inversely proportional to the measured CO_2_ concentration (Fig. 2). This trend is also confirmed by a highly significant linear regression model (R^2^ = 0.80, p < 0.001).

### Mass balance calculations and the apportionment of CO_2_ contributions

**Figure 3:**
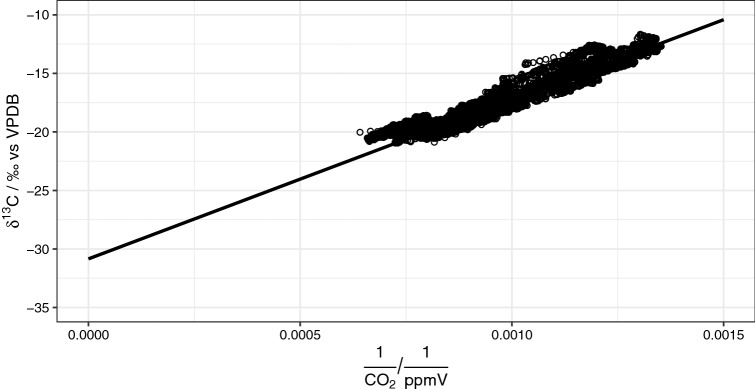
Keeling plot with linear regression indicating an intercept of − 30.8 ± 0.05‰ as end-member for the combustion-related CO_2_-emissions.

Concentrations of geogenic CO_2_ were determined based on a mass balance calculation that assumes a mixture between three end-members. These are (1) fresh air, (2) CO_2_ from combustion, and (3) geogenic CO_2_, with average isotope ratios of − 8.9‰, − 30.8‰, and − 6.0‰, respectively. While the end-member for fresh air was obtained from the literature^28^, the value for combustion was determined from a Keeling plot^29^. Briefly, the Keeling plot technique uses the linear regression of the isotope composition against the inverse of its concentration to approximate the isotope value for a theoretical infinite concentration at the intercept^30^. With this technique, an intercept of − 30.8 ± 0.05‰ was determined for *δ*^13^C-CO_2_ (Fig. 3) that is well within the range of direct measurements of exhaust from machinery used within the mine. These lay between − 31.3 and − 28.8‰ with an average of − 29.8 ± 0.8‰; based on five different cars, four different models, and five replicates each.

The isotopic composition of geogenic CO_2_ was characterized by discrete measurements at a location in the mine, where high concentrations (99%) of geogenic CO_2_ are stripped from a cavernous structure (*i.e.,* fault-zone). These high concentrations and it’s similarity to published isotopic values of geogenic CO_2_ support the choice as end-member in this study^1,31,32^.

An isotopic balance equation

1 $$\delta^{13} C_{T} \times C_{T} = \delta^{13} C_{F} \times C_{F} + \delta^{13} C_{C} \times C_{C} + \delta^{13} C_{G} \times C_{G}$$

where C_T_ represents the total concentration of CO_2_ while C with the subscripts G, F, and C represent the relative contributions of geogenic, fresh air, and combustion-related sources. C_T_ was measured, C_F_ was assumed to be 400 ppmV, thereby reducing a three-endmember mixing model to a two-member mixing model.

By assuming that

2 $$C_{T} = C_{G} + C_{F} , + C_{C} ,$$

we were able to replace C_C_ in Eq. (1) by substitution. This led to the following mass-balance equation:

3 $$C_{G} = \left( {\delta^{13} C_{T} \times \, C_{T} - \, \delta^{13} C_{F} \times \, C_{F} - \, \delta^{13} C_{C} \times \, C_{T} + \, \delta^{13} C_{C} \times \, C_{F} } \right) \, /\left( {\delta^{13} C_{G} - \, \delta^{13} C_{C} } \right)$$

Therefore, if the total concentration is measured, the relative contributions of each source can be calculated. A classical error propagation^33,34^ was employed to obtain the level of uncertainty for the mixing-model-derived geogenic CO_2_ contribution. A conservative estimate of the uncertainty (Table 1) was chosen for all components except the measured total CO_2_, which was derived from performance tests done in the lab.

**Table 1:** Values and uncertainties of end-members used in the mixing model.

Source of CO_2_	Concentration / ppmV	*δ*^13^C / ‰ vs VPDB*
Atmosphere	400 ± 10	− 8.9 ± 0.2
Geology	Calculated	− 6 ± 1
Combustion	Calculated	− 30.8 ± 1.0
Total CO_2_	Measured ± 10	Measured ± 0.2

*Vienna Pee-Dee Belemnite is an internationally recognized carbon isotope standard that is described in the “Methods” section.

By application of Eq. (3), combustion was determined to add between 128 to 854 ppmV CO_2_. Thus, contributing between 17 and 57% of the CO_2_ present throughout the long-term measurement. The mean contribution of this end-member was 45%.

### Geogenic CO_2_ dynamics

**Figure 4:**
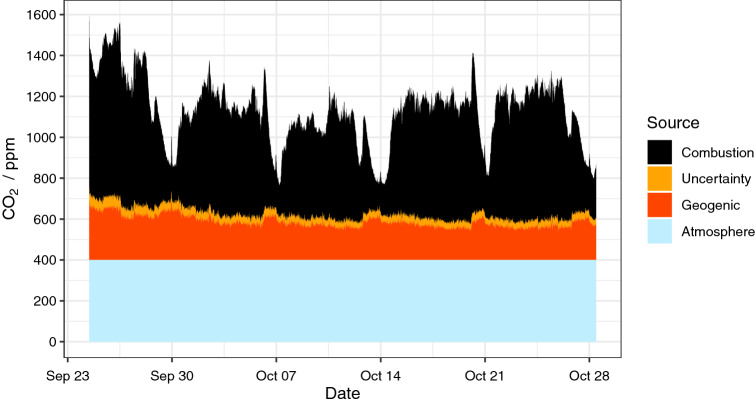
Concentrations and composition of CO_2_ according to the mixing model. Tick marks on x-axis indicate Mondays. The range of uncertainty was obtained via error propagation from all the sources (Table 1).

Interestingly, the mean contribution of geogenic CO_2_ was generally lower, with an average 18% contribution of the total present CO2 and a smaller spread ranging between 11 and 32% contribution. Thus, with an average contribution of 194 ppmV, smaller but noticeable variations that ranged between 137 and 324 ppmV over the entire measurement period were observed. Noticeable increases in the geogenic contribution coincide with substantial decreases in the total CO_2_ concentration present. (Fig. 4). These increases in geogenic CO_2_ do not seem to be instantaneous and do not follow a simple linear correlation to the overall concentration. Nevertheless, 28% of the variation within the geogenic CO_2_-contributions can be explained by a highly significant negative correlation to the difference between the daily and weekly moving average of the total CO_2_-concentration (ranged major axis, R^2^ = 0.283, p < 0.01, [CO_2_]_geo_ = − 0.180 * *Δ*CO_2_ + 185.5). This means that geogenic CO_2_ contributions increased when overall CO_2_ concentrations were lowered over an extended period.

Variance partitioning via partial regression of the total CO_2_ variability suggests that the most substantial part of the variance (*i.e.,* 92%) is related to combustion. Of the remaining 8% variation ascribed to geogenic CO_2_ fluctuations, approximately 5% are attributed to an interactive term. This indicates that most of the variation observed in geogenic concentration was related to changes in the combustion-related CO_2_.

## Discussion

Repeating weekly patterns are visually recognizable and were confirmed by autocorrelation with a 7-day lag. They represent two-thirds of the variation within the time series. These patterns result from internal mine-traffic and stationary combustion-related mine-operations that also follow a weekly pattern with work and weekend-shifts.

Carbon released from fossil fuels via combustion is ultimately of photosynthetic origin. Thus, resulting *δ*^13^C values are similar to values typically found for C_3_-plants and range between − 38 and − 24‰^30,35^. Recently published values for gasoline *δ*^13^C_CO2_ exhaust range around − 28.7‰^36^. Due to its integrative nature of all the machinery used in the mine, we consider the value determined via the Keeling plot technique (− 30.8 ± 0.05‰) being most robust; it also lays well within the expected range from literature and own discrete measurements.

Geogenic CO_2_ is characterized by *δ*^13^C values ranging between − 7 and + 2‰^1,31,32^. Thus, it significantly differs from fossil fuel isotopic compositions. The contact with water accessing into the geological faults may have led to isotopic exchange processes with especially the oxygen atoms of the encountered CO_2_, why, in this study, we focus on carbon isotopes. A repeated solution-degassing cycle might alter the isotopic C signal through fractionation^37–39^. We have no means to investigate this further due to lacking information on the constituent geological features. In this study, we had access to the source signal, which was determined at the outflow of the geological fault with a value of − 6‰ and a CO_2_ concentration of 99 vol%.

The isotopic value of ^13^C–CO_2_ in the atmosphere has decreased considerably within the past 200 years due to an increasing proportion of fossil-fuel-derived CO_2_ in the atmosphere and has decreased from preindustrial − 6.5‰ to values close to − 9‰^28,40,41^. For this study, we assume an isotope value of fresh air ranging around − 8.9‰ that was obtained by linear approximation from the literature^28^. For simplicity, we have assumed a constant 400 ppmV as the atmospheric CO_2_ baseline. In temperate climate regions, mean diurnal variations in rural areas tend to be below 20 ppmV^42,43^. Compared to the daily variations detected in the mine that ranged between 67 and 591 ppmV (average: 223 ppmV), the existing natural variabilities are negligible. However, in more populated areas, daily variations in CO_2_ may exceed 100 ppmV due to the combustion of fossil fuels above ground in cities^44–47^. In this respect, the mixing model (Eq. (3)) is not able to differentiate between external and internal traffic of the salt mine. Nonetheless, the ventilation air intake is placed in a predominantly rural area with little traffic. We can, therefore, assume the first scenario with negligible variations of the atmospheric input.

Contributions of a relatively stable geogenic CO_2_ influx are inversely correlated to changes in the total CO_2_ concentration, as shown in Fig. 4 and corroborated using variance partitioning, indicating that roughly 63% of the variation in the geogenic proportion was linked to variations in the combustion-related CO_2_ emissions. Ranged major axis correlation indicates that the short term (daily) established CO_2_ concentrations in the mine are negatively correlated to the geogenic-derived CO_2_ proportions. Exact boundary conditions (i.e., surface area, total volume, fluctuations of air pressure, and the exchange rate of the passing gas) were not available for this study due to confidentiality agreements with the mine operator. Thus, it was neither possible to quantify the exact exchange rates, nor total amounts, nor fluxes of CO_2_ released over the entire mine. However, as the instrument was placed in direct vicinity of a continuous mine air extraction system with a known flow rate, it was possible to estimate an average contribution of 112 tons of geogenic CO_2_ per year (194 ppm × 600 m^3^ min^−1^) for the single location monitored.

Furthermore, our work shows that the migration of geogenic CO_2_ into the active salt-mine relates to established concentration gradients, indicating that the presence of combustion CO_2_ attenuates the release of geological CO_2_. This observation follows the well-established Fick’s law^48^ and is one of the first of such observations in a mine. Temperature-changes due to the absence of machine-induced heat during the weekend would influence the concentration gradient further based on general thermodynamic laws. A lower temperature would, thus, induce a higher diffusive transport from geogenic CO_2_ into the mine. However, the continuous ventilation should ensure fairly constant climatic conditions. In combination with the rather large size of the mine, this should reduce temperature-related effects on diffusion.

The dominance of combustion-related CO_2_ may have implications on ventilation efficiencies of below-ground mining operations, as described below. This study has identified lagged responses of machine-derived CO_2_ after its reduced emissions due to reduced mining operations over the weekends. A period of about one day preceded the first significant concentration decrease found on Saturdays. Similarly, Mondays, when work had already been taken up again, always exhibited the lowest overall concentrations. In part, this pattern finds its explanation in the 24-h interval applied as the standard day observation interval. However, this time lag also serves as a measure for the efficiency of the mine ventilation system. Therefore, the current measurements may be used as a proxy for the necessary exchange time needed to reduce unwanted gases by about two thirds.

The fact that the combustion end-member was most dominant and assumed a mean of 45% over the observation period also implies a low degree of geogenic input and a higher degree of safety at this location. This may be different at other locations where geogenic inputs are higher. While CO_2_ inputs by machinery might also be determined by fuel consumption in the mine, geogenic contributions demand more sophisticated techniques such as the ones presented above.

Note that while the relative composition of the three end-members was determined at one location and proportions may vary significantly throughout the mine, the signal of geogenic CO_2_ integrates over the entire flow path from the fresh air influx to the extraction ventilation. To establish exact emission-rates through a defined area of the mine, a timed array of sampling-locations that resolves incremental increases along the flow of the mine aeration or measurements at in and outflow locations would have been necessary. While this would be an additional task for future investigations, this study at a single location lays the fundamentals for such follow-up studies and demonstrates the benefit of long-term isotope observations to quantify dynamic variabilities of total CO_2_ and its apportionment into different sources. In volcanic influenced areas, extensive monitoring has already proven beneficiary to estimate total carbon fluxes into the atmosphere^8,49^, and similar studies should be extended to areas where geogenic CO_2_ emissions are suspected. Mine venting systems provide an opportunity for these kinds of investigations when mine topology and ventilation parameters can be obtained.

## Conclusions

The presented high-resolution measurements, in combination with a long-term period, revealed so far unknown dynamics and interactions within the atmosphere of an active salt mine. Variations of the total CO_2_ coincided with the weekly operation of the mine. Mass balance calculations, based on isotopically distinct end-members, show that the use of machinery represents the largest and most variable contribution to the overall ambient CO_2_ concentrations. Our data suggest that changes in resultant concentration gradients were predominantly responsible for variations in geogenic CO_2_ contributions.

The applied technique may help outline variations that may be operational, such as shown here, or depend on diurnal or seasonal factors. Importantly, it can also transfer to other environments in which CO_2_ dynamics play a crucial role—including soils, caves, or industrial applications.

## Materials and methods

### Location

The geology of the salt deposit at a depth of 600 m below ground is characterized by late Permian Paleozoic evaporitic sediments of the Zechstein-group^50^. Highly carbonated, gas-bearing saline waters were encountered by mining operations at a geological fault-zone in the late 1950s. Analyses of the gas-phase within this study indicate a CO_2_-dominated composition (99% vol) determined by means of a quadrupole mass spectrometer (OmniStar, Pfeiffer Vacuum, Germany).

A site approximately 1 km east of this fault, where contributions of geogenic CO_2_ were observed in the past, was chosen for long-term measurements from Tuesday, September 24, to Monday, October 28, 2019. The location was situated in a dead-end tunnel, about 3 km away from the mine's entrance shaft, and well vented via a continuous used-gas-extraction-ventilation system (600 m^3^ min^−1^) in close proximity (5 m) of the instrument’s sample intake.

### Instrumentation

An isotope ratio mid-infrared laser spectrometer (Delta Ray, Thermo Scientific, Bremen, Germany) was used to measure CO_2_ concentrations and its stable isotope ratios of ^13^C/^12^C. The instrument is also capable of measuring ^18^O/^16^O ratios of CO_2_; however, for the sake of focus and various exchange processes that may influence its oxygen isotopes (including interactions with water), this manuscript only discusses the carbon data.

For isotope determinations, mine air was pumped into the laser cavity of the spectrometer at a continuous flow rate of 80 mL per minute via a steel capillary (I.D. 0.16 mm) of about 10 m length that was placed directly at the wall of the mine in approximately 160 cm height. The instrument was set to continuous measurement mode that yielded measurements every second. Concentration measurements of CO_2_ relied on the instrument's internal calibrations verified with a 1000-ppmV standard in a synthetic air mixture (Air Liquide Deutschland, Düsseldorf, Germany).

Internal corrections for concentration-related shifts and delta-scale contraction were applied according to the manufacturer’s recommendations. To ensure measurement-stability, the instrument was calibrated against a working standard with a known isotope value of *δ*^13^C–CO_2_ = − 34.3‰ for 5 min every hour. Setup of the reference gas containers, valves, and calibration procedures are described in the instrument’s handbook. The working standard's isotopic value was beforehand established by means of dual-inlet-IRMS measurements (Delta V plus, Thermo Fisher Scientific, Bremen, Germany) against an internationally certified standard (RM8563, NIST, Gaithersburg, MD, USA). The first two minutes of each standard working cycle were discarded in order to flush the system. Subsequently, the raw measurements were recorded every second and averaged over three-minute intervals to provide a single value of the working standard for calibration. When switching back to the measurement of ambient CO_2_, the first 2 min were also discarded, again as a system flush.

### Isotope notation

All ^13^C/^12^C ratios are reported using the *δ*-notation against the internationally recognized ratio of Vienna Pee-Dee Belemnite (VPDB) and expressed in permille (‰)^51,52^:

$$\delta^{{{13}}} {\text{C }} = \left( {R_{{{\text{sample}}}} /R_{{{\text{standard}}}} - {1}} \right)$$

where *R* represents the ratio of the heavier to the most abundant stable isotope of the corresponding element (e.g., *R* = ^13^C/^12^C), with ^13^*R*_VPDB_ = 0.0111802^53^, as recorded in the Delta Ray software (Qtegra, version 2.5.2198.57, Thermo Fisher Scientific, Bremen, Germany).

### Statistical analysis

All statistical analyses were performed with the statistical program R^54^. Analysis of variance (ANOVA) is one of the most commonly used statistical techniques to test the significance of differences between means of independent classes^55^. It can be viewed as an extension of a two-sample t-test and is based on the partitioning of the total sum of squared errors (variance) into the relevant classes. Thus, ANOVA requires normal distribution and non-significant differences between the variance for each class (homoscedasticity). So-called post hoc tests are necessary to establish the exact order and the significance level between the different classes after an established significant ANOVA. Tukey’s honest significant difference (HSD) was applied, where necessary^56^.

Time series were decomposed into seasonal, random, and trend components by using a classical additive decomposition approach^27^ implemented in the ‘stats’ package 3.2.6. of R^54^. Seasonal time intervals were visually identified and verified by computation of estimates of the autocorrelation function (*i.e.,* a linearly related but lagged response variable) as defined in Venables and Ripley^57^ and implemented in the ‘stats’ package 3.2.6. of R^54^. To partition the concentration variation observed, a partial linear regression model approach, as described in Borcard, et al.^58^ and implemented in vegan package 2.5–6^59^, was used.

## Data Availability

Data is freely available on the Pangea server (www.pangaea.de) and cited in the references^60^.

## References

[1] Chiodini G (2004). Carbon dioxide Earth degassing and seismogenesis in central and southern Italy. Geophys. Res. Lett..

[2] Mörner N-A, Etiope G (2002). Carbon degassing from the lithosphere. Glob. Planet. Change.

[3] Irwin WP, Barnes I (1980). Tectonic relations of carbon dioxide discharges and earthquakes. J. Geophys. Res. Solid Earth.

[4] Tamburello G, Pondrelli S, Chiodini G, Rouwet D (2018). Global-scale control of extensional tectonics on CO_2_ earth degassing. Nat. Commun..

[5] Camarda M, De Gregorio S, Di Martino RMR, Favara R (2016). Temporal and spatial correlations between soil CO2 flux and crustal stress. J. Geophys. Res. Solid Earth.

[6] Di Martino RMR, Capasso G, Camarda M (2016). Spatial domain analysis of carbon dioxide from soils on Vulcano Island: Implications for CO2 output evaluation. Chem. Geol..

[7] Camarda M (2019). The monitoring of natural soil CO2 emissions: Issues and perspectives. Earth Sci. Rev..

[8] Di Martino RMR, Capasso G, Camarda M, De Gregorio S, Prano V (2020). Deep CO2 release revealed by stable isotope and diffuse degassing surveys at Vulcano (Aeolian Islands) in 2015–2018. J. Volcanol. Geotherm. Res..

[9] Camarda M, De Gregorio S, Di Martino R, Favara R, Prano V (2020). Relationship between soil CO2 flux and tectonic structures in SW Sicily. Ann. Geophys..

[10] Chiodini G (2020). Correlation between tectonic CO2 Earth degassing and seismicity is revealed by a 10-year record in the Apennines, Italy. Sci. Adv..

[11] Rey A (2015). Mind the gap: non-biological processes contributing to soil CO_2_ efflux. Glob. Chang. Biol..

[12] Rey A, Etiope G, Belelli-Marchesini L, Papale D, Valentini R (2012). Geologic carbon sources may confound ecosystem carbon balance estimates: Evidence from a semiarid steppe in the southeast of Spain. J. Geophys. Res. Biogeosci..

[13] Burton MR, Sawyer GM, Granieri D (2013). Deep carbon emissions from volcanoes. Rev. Mineral. Geochem..

[14] Viveiros F (2020). Deep CO2 emitted at Furnas do Enxofre geothermal area (Terceira Island, Azores archipelago). An approach for determining CO2 sources and total emissions using carbon isotopic data. J. Volcanol. Geotherm. Res..

[15] Di Martino RMR, Camarda M, Gurrieri S, Valenza M (2013). Continuous monitoring of hydrogen and carbon dioxide at Mt Etna. Chem. Geol..

[16] Liuzzo M, Gurrieri S, Giudice G, Giuffrida G (2013). Ten years of soil CO2 continuous monitoring on Mt. Etna: exploring the relationship between processes of soil degassing and volcanic activity. Geochem. Geophys. Geosyst..

[17] Rizzo AL (2014). Real-time measurements of the concentration and isotope composition of atmospheric and volcanic CO_2_ at Mount Etna (Italy). Geophys. Res. Lett..

[18] Granieri D, Chiodini G, Marzocchi W, Avino R (2003). Continuous monitoring of CO_2_ soil diffuse degassing at Phlegraean Fields (Italy): influence of environmental and volcanic parameters. Earth Planet. Sci. Lett..

[19] Bini G (2020). Deep versus shallow sources of CO_2_ and Rn from a multi-parametric approach: the case of the Nisyros caldera (Aegean Arc, Greece). Sci. Rep..

[20] Lucic G, Stix J, Wing B (2015). Structural controls on the emission of magmatic carbon dioxide gas, Long Valley Caldera, USA. J. Geophys. Res. Solid Earth.

[21] Capasso G, Di Martino RMR, Camarda M, Prano V (2017). Dissolved carbon in groundwater versus gas emissions from the soil: the two sides of the same coin. Procedia Earth Planet Sci..

[22] Dietzel M, Schwecke H, Hirschfeld A, Röhring M, Böttcher ME (1997). Geochemical and ^13^C/^12^C-isotopical investigation of mineral waters in northern Hessia (Germany) and the origin of their CO_2_ Content. Acta Hydrochim. Hydrobiol..

[23] Hedlund FH (2012). The extreme carbon dioxide outburst at the Menzengraben potash mine 7 July 1953. Saf. Sci..

[24] Mayer B (2015). Assessing the usefulness of the isotopic composition of CO_2_ for leakage monitoring at CO_2_ storage sites: a review. Int. J. Greenh. Gas Control..

[25] Bogaard PJF (2003). Petrogenesis of basanitic to tholeiitic volcanic rocks from the Miocene Vogelsberg, central Germany. J. Petrol..

[26] Ehrenberg KH (1981). Forschungsbohrungen im Hohen Vogelsberg (Hessen) Bohrung 1 (Flösser-Schneise), Bohrung 2/2A (Hasselborn). Geol. Abhandlungen Hessen.

[27] Kendall M, Stuart A, Ord J (1983). The Advanced Theory of Statistics.

[28] Graven H (2017). Compiled records of carbon isotopes in atmospheric CO_2_ for historical simulations in CMIP6.

[29] Keeling CD (1958). The concentration and isotopic abundances of atmospheric carbon dioxide in rural areas. Geochim. Cosmochim. Acta.

[30] Pataki DE (2003). The application and interpretation of Keeling plots in terrestrial carbon cycle research. Glob. Biogeochem. Cycles.

[31] Bräuer K, Kämpf H, Niedermann S, Strauch G, Tesař J (2008). Natural laboratory NW Bohemia: comprehensive fluid studies between 1992 and 2005 used to trace geodynamic processes. Geochem. Geophys. Geosyst..

[32] Apps JA, Van de Kamp PC (1993). Energy gases of abiogenic origin in the Earth’s crust. U.S. Geol. Surv. Prof. Paper.

[33] Taylor JR (1997). Propagation of Uncertainties. Introduction to Error Analysis, The Study of Uncertainties in Physical Measurements.

[34] Clifford AA (1973). Multivariate Error Analysis: A Handbook of Error Propagation and Calculation in Many-Parameter Systems.

[35] Jenden PD, Hilton DR, Kaplan IR, Craig H (1993). Abiogenic hydrocarbons and mantle helium in oil and gas fields. U.S. Geol. Surv. Prof. Paper.

[36] Schumacher M (2011). Oxygen Isotopic Signature of CO_2_ from Combustion Processes.

[37] Mook WG, Bommerson JC, Staverman WH (1974). Carbon isotope fractionation between dissolved bicarbonate and gaseous carbon dioxide. Earth Planet. Sci. Lett..

[38] Clark I, Fritz P (1997). Tracing the Carbon Cycle. Environmental Isotopes in Hydrogeology.

[39] Myrttinen A, Becker V, Barth JAC (2012). A review of methods used for equilibrium isotope fractionation investigations between dissolved inorganic carbon and CO2. Earth Sci. Rev..

[40] Francey RJ (2016). A 1000-year high precision record of δ13C in atmospheric CO2. Tellus B.

[41] Yakir D, Holland HD, Turekian KK (2003). The stable isotopic composition of atmospheric CO_2_. Treatise on Geochemistry.

[42] Imasu R, Tanabe Y (2018). Diurnal and seasonal variations of carbon dioxide (CO_2_) concentration in urban, suburban, and rural areas around Tokyo. Atmosphere.

[43] Murayama S (2003). Temporal variations of atmospheric CO_2_ concentration in a temperate deciduous forest in central Japan. Tellus B.

[44] Newman S, Xu X, Affek HP, Stolper E, Epstein S (2008). Changes in mixing ratio and isotopic composition of CO_2_ in urban air from the Los Angeles basin, California, between 1972 and 2003. J. Geophys. Res..

[45] Widory D, Javoy M (2003). The carbon isotope composition of atmospheric CO 2 in Paris. Earth Planet. Sci. Lett..

[46] Górka M, Lewicka-Szczebak D (2013). One-year spatial and temporal monitoring of concentration and carbon isotopic composition of atmospheric CO2 in a Wrocław (SW Poland) city area. Appl. Geochem..

[47] Idso SB, Idso CD, Balling RC (2002). Seasonal and diurnal variations of near-surface atmospheric CO_2_ concentration within a residential sector of the urban CO_2_ dome of Phoenix, AZ, USA. Atmos. Environ..

[48] Fick A (1855). Ueber diffusion. Ann. Phys..

[49] Venturi S (2019). A multi-instrumental geochemical approach to assess the environmental impact of CO2-rich gas emissions in a densely populated area: The case of Cava dei Selci (Latium, Italy). Appl. Geochem..

[50] Richter-Bernburg G (1953). Über salinare Sedimentation. Z. Dtsch. Geol. Ges..

[51] McKinney CR, McCrea JM, Epstein S, Allen HA, Urey HC (1950). Improvements in mass spectrometers for the measurement of small differences in isotope abundance ratios. Rev. Sci. Instrum..

[52] Coplen TB (2011). Guidelines and recommended terms for expression of stable-isotope-ratio and gas-ratio measurement results. Rapid Commun. Mass Spectrom..

[53] Werner RA, Brand WA (2001). Referencing strategies and techniques in stable isotope ratio analysis. Rapid Commun. Mass Spectrom..

[54] R Core Team. *R: A Language and Environment for Statistical Computing v. 4.0.0 R Foundation for Statistical Computing* https://www.R-project.org (2020).

[55] Fisher RA, Kotz S, Johnson NL (1992). Statistical methods for research workers. Breakthroughs in statistics.

[56] Tukey JW (1949). Comparing individual means in the analysis of variance. Biometrics.

[57] Venables W, Ripley B (2002). Time Series Analysis. Modern Applied Statistics with S.

[58] Borcard D, Legendre P, Drapeau P (1992). Partialling out the spatial component of ecological variation. Ecology.

[59] Oksanen, J. *et al.**vegan: Community Ecology Package v. 2.5-6. *https://CRAN.R-project.org/package=vegan (2019).

[60] Frank, A. H. *et al.* Long-term and high-temporal-resolution δ^13^C-CO_2_ in a salt mine. *PANGAEA*, https://doi.pangaea.de/10.1594/PANGAEA.917467 (2020).

